# Stability, homogeneity and measurement uncertainty estimation of PVP-I solutions for the application on oro and nasopharynx against SARS-CoV-2

**DOI:** 10.1038/s41598-024-52346-3

**Published:** 2024-03-27

**Authors:** Md. Moniruzzaman, Mamudul Hasan Razu, Sad Al Rezwan Rahman, Nayan Kumer Kundu, Sabiha Kamal, Mala Khan

**Affiliations:** Bangladesh Reference Institute for Chemical Measurements (BRiCM), Dr-Qudrat-I-Khuda Road, Dhanmondi, Dhaka, 1205 Bangladesh

**Keywords:** Virology, SARS-CoV-2

## Abstract

Aqueous solution containing different concentration (0.5, 0.6 and 1.0%) (w/v) of Polyvinyl pyrrolodon-Iodine (PVP-I) complex, a well-known antiseptic; is prepared and the stability and homogeneity of these solution is assessed as per the ICH Guidelines and International Harmonized Protocol respectively. The solutions were found to be sufficiently homogeneous and stable for a year at 25 °C (60%RH). Measurement uncertainty of the prepared PVP-I solutions were estimated by identifying possible sources of uncertainty using Ishikawa diagram and preparing uncertainty budget based on scope of calibration laboratory. The stable and homogenized PVP-I solution is to be used in a clinical trial for the application on oro and nasopharynx against novel SARS-CoV-2 Virus.

## Introduction

In the late 2019 the unexpected inception of COVID-19 commenced due to novel coronavirus, SARS-CoV-2; and has been declared as pandemic by WHO because of its nature and severity. Though vaccination program has already been implemented in many countries, it will take a long time to vaccinate this huge world population. But concern still remains whether this global vaccination program declines the exposure or affected rate^1^. Hence precautionary measures like social distancing, lock down, personal hygiene, testing, contact tracing, and universal masking are still in effect worldwide. But issues like material of construction, uses and disposal practice of face masks have raised questions on sustainable environmental point of view^2,3^. Therefore an additional preventive measure alongside with mask, sanitization and social distancing should be taken until all of the population is being vaccinated or any other sustainable solution appears.

So far it has been known that the nasal cavity, oropharynx and nasopharynx are the primary target cell of novel corona virus SARS-CoV-2^4–6^ where the host cell becomes infected due to interaction between viral spike protein (SP) and host cell receptor Angiotensin Converting Enzyme 2 (ACE_2_) or Basigen/EMMPRIN (CD_147_)^7^. Viral Access and contamination of the mentioned pathway can be prevented by wearing mask but the infection incidence, if happens, despite wearing mask or not, can be reduced upon applying an effective antiseptic agent that is able in preventing viral colonization or load. Povidone-iodine, a stable chemical complex of polyvinylpyrrolidone (PVP), is an established antiseptic with a broad spectrum used extensively from 0.5 to 10% in mouth wash, hand wash, skin preparation, ophthalmic surgery and oral surgery^8–11^. The antiseptic action of PVP-I starts when free iodine dissociates from the polymer complex that rapidly penetrates the microbes and oxidizes the nucleic acid and its protein structures^12^. Several reports have shown that, not only active in vitro against SARS-COV (epidemic of 2002–03) & MERS-COV (epidemic of 2012–13) virus, but also against SARS-CoV-2 virus^13–16^.

Stability of a pharmaceutical product is defined as the ability of a particular formulation in a specific container/enclosure system to remain within its physical, chemical, microbiological, toxicological, protective and informational specifications^17^. In other words, it is the extent of a product to remain within its specification from packaging to use. Stability testing assesses the effect of environmental factors on the quality of the drug substance or a formulated product that is utilized to estimate its shelf life; appropriate storage conditions and suggests labeling instructions. Furthermore, the data produced during the stability testing is a crucial requirement for regulatory approval of any drug or formulation^18^. On the other hand, homogeneity is defined as the degree of uniformity distributed throughout a quantity of a material or solution. Drug product homogeneity defines the sameness of quality attribute(s) across an entire batch. From product quality and regulatory perspective, homogeneity within a batch and consistency between manufactured batches are keys to safeguard public health against numerous sources of variability^19^. Furthermore, quality control (QC) testing performed during release and stability studies demands homogeneity assessment of product batches for the justification of sample size used^19^.

Uncertainty is usually defined as the parameter associated with the measurement which illustrates reasonably attributed value dispersions of the measurand^20^. Every measurement has a degree of uncertainty irrespective of precision and accuracy. This is caused by two factors, the measuring instrument limitation (systematic error) and the personnel`s skill performing the measurements (random error). Estimation of uncertainties associated with results helps experts to observe their findings more precisely. It is important to compare results between different laboratories or from the same laboratory at different times, with confidence. This can be attained by ensuring the same ‘reference points’ used by all laboratories through establishing a chain of calibrations leading to primary national or international standards, ideally the Systeme Internationale (SI) units of measurement. This unbroken chain of comparisons leading to a known reference value provides ‘traceability’ to a common reference point, ensuring that different experimenters are using the same measurement units. Traceability is therefore closely connected to uncertainty. Traceability provides the means of placing all related measurements on a consistent measurement scale, whereas uncertainty characterizes the ‘strength’ of the links in the chain and the agreement to be expected between laboratories performing similar measurements^21^.

Hence, the purpose of this study is to assess the homogeneity and stability of prepared different concentration of PVP-I solution which will be used in a clinical trial for the application in on oro-nasopharings against novel SARS-CoV-2 Virus. This study alongside successful completion of clinical trial will provide an advantage to the pharmaceutical industries regarding the shelf life of the product and regulatory approval as well. Uncertainty estimation will ensure traceability up to SI unit from metrological aspect.

## Materials & methods

### Chemicals

All chemicals and reagents were USP or ACS grade and were used as received basis. PVP-I (purity as available iodine: 11.2%), acetic acid (purity: 99.7%), starch (pH of 2% solution: 6.0–7.5), sodium thiosulfate (purity: 99.5%) was obtained from Sigma-Aldrich (Germany) and glycerin (purity: 99.5%) was supplied by Merck (Germany). Sterile water was used in preparation of different concentration of PVP-I solution. Deionized water (pH 5.65; conductivity < 2.0 μS/cm), produced from own plant of BRiCM was used in all other purpose unless mentioned otherwise.

### Equipment

All pH values were measured by Thermo Scientific, USA pH meter (Model: Orion Star A215), weighing were performed using Mettler Toledo, Switzerland electrical Laboratory balance (Model: AL204) and absorbance were measured by Shimadzu, Japan UV–Visible Spectrophotometer (Model: UV-1800). Sterile water were produced in Perlong, China vertical pressure steam sterilizer (Model: PTS-B50L) at 120 °C and 15lbs for 20 min.

### Preparation of different concentration of PVP-I solution

0.5% (w/v), 0.6% (w/v) and 1.0% (w/v) PVP-I solution were prepared by sterile water in a dark place. Formulation of different concentration of PVP-I solution is shown in Table 1. Prepared solutions were then bottled in 20 mL PTFE amber colored oro-nasal spray bottle for stability & homogeneity assessment and for further application on oro-nasopharings against novel SARS-CoV-2 Virus.

**Table 1:** Formulation of different concentration of PVP-I solution.

	PVP-I (%) (w/v)	Glycerin (%) (v/v)
0.5% (w/v) PVP-I	0.5	16.7
0.6% (w/v) PVP- I	0.6	16.7
1.0% (w/v) PVP- I	1.0	16.7

### Determination of available iodine

Available iodine in the prepared solution were determined according the official analytical method of the USP. Briefly, 1 mL sample solution added to 150 mL deionized water and stirred for 1 h in dark. 0.1 mL dilute acetic acid added and titrated with 0.1 M sodium thiosulfate using starch solution as indicator. 1 mL of 0.1 M sodium thiosulfate is equivalent to 12.69 mg of available iodine.

### Measurement of absorbance at 368 nm

Prepared PVP-I solution were first diluted 10times by deionized water and then measured at 368 nm against deionized water as reference.

### Homogeneity assessment

Homogeneity of different concentration of PVP-I solution were assessed at different storage conditions i.e. 25 °C (60%RH), 30 °C (65%RH) and 40 °C (75%RH) on the following day of formulation. A set of 10 oro-nosal spray bottle from each formulation were randomly selected. Each sample was analyzed in duplicate. Statistical analysis like Cochran Test on pH, available iodine and absorbance of the PVP-I solutions was executed for homogeneity assessment^22^.

### Stability assessment

Stability of prepared PVP-I solutions were assessed at 25 °C (60%RH), 30 °C (65%RH) and 40 °C (75%RH) for the period of 12 months^23,24^. Same set of oro-nosal spray bottle that were used for homogeneity assessment were also used for this purpose. pH, available iodine and absorbance of the PVP-I solutions were measured at 2 months interval. The mean values of results were used for statistical analysis to establish a linear regression and statistical significance were investigated using one way ANOVA^22,25^.

### Measurement uncertainty estimation

Following flow chart will be helpful in estimating measurement uncertainty.
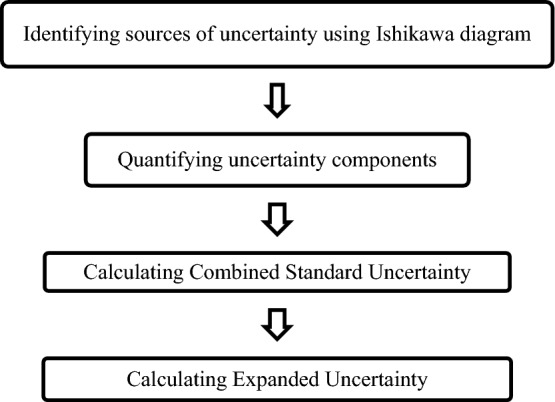


Ishikawa diagram is constructed for the parameter pH, available iodine and absorbance to find out possible sources of uncertainty. Uncertainty from balance, volumetric flask, measuring cylinder, burette, pH meter, homogeneity, stability and repeatability were identified. The value of uncertainty arising from balance, volumetric flask, measuring cylinder, burette and pH meter were obtained from respective calibration certificate. Standard deviation of repeatability and stability were directly considered as standard uncertainty. The estimative of standard uncertainty due to homogeneity (*u*_*hom*_) was calculated by Eq. (1)^26^

1 $$u_{hom} = \sqrt {\frac{{MS_{within} }}{n}} \cdot \sqrt[4]{{\frac{2}{{f_{within} }}}}$$

where *MS*_*within*_ represents the mean square within groups of one way ANNOVA, *f*_*within*_ represents degree of freedom of *MS*_*within*_ one way ANNOVA and *n* represents number of replicates.

Once all the uncertainty components are quantified, the combined standard uncertainty (*u*_*c*_) is calculated using Eq. (2)^21^

2 $$u_{c} = \sqrt {\sum \left( {\frac{{u_{xi} }}{xi}} \right)^{2} }$$

where *u*_*xi*_*/xi* represents uncertainties of each componenet expressed as relative standard deviation.

Finally the expanded uncertainty (*U*) is calculated using Eq. (3)

3 $$U = k \, * \, u_{c}$$

where *k* = 2, the coverage factor at 95% confidence interval.

## Results & discussions

### Homogeneity of different concentration of PVP-I solution

The homogeneity of formulated PVP-I solutions can be understood by performing Cochran test. Cochran`s test statistic is calculated and compared with the appropriate critical value. If the *C*_*Cochran*_ < *C*_*Critical*_; then the solution is considered to be homogeneous^21^. The critical values of 7–20 pairs at 95% confidence level are mentioned in Table 2. Cochran’s test statistic is calculated by following equation;

$${C}_{Cochran}= \frac{{D}_{max}^{2}}{\sum_{m}{D}_{i}^{2}}$$

where ${D}_{i}^{2}$ = squared difference of each pair, ${D}_{max}^{2}$ = maximum squared difference, ${C}_{Cochran}$= Cochran test statistic.

**Table 2:** Critical values for Cochran test statistics.

Pairs	7	8	9	10	11	12	13	14	15	16	17	18	19	20
95%	0.727	0.68	0.638	0.602	0.57	0.541	0.515	0.492	0.471	0.452	0.434	0.418	0.403	0.389

The calculated Cochran test statistic for pH, available iodine and absorbance of 0.5%, 0.6% and 1.0% PVP-I solutions at storage conditions i.e. 25 °C (60%RH), 30 °C (65%RH) and 40 °C (75%RH) is tabulated in Table 3.

The critical value for 10 pair is found to be 0.602 from Table 2. Since all of calculated *C*_*Cochran*_ < *C*_*Critical*_; it can be said that all the prepared PVP-I solution (0.5%, 0.6% and 1.0%) is homogeneous at all of the studied storage conditions (Table 3). An example for calculating the Cochran’s test statistic of pH of 0.6% PVP-I solution at storage conditions 25 °C (60%RH) is illustrated below in Table 4.

**Table 3:** Calculated Cochran test statistic of different concentration of PVP-I solution.

	0.5% PVP-I			0.6% PVP-I			1.0% PVP-I		
	25 °C	30 °C	40 °C	25 °C	30 °C	40 °C	25 °C	30 °C	40 °C
pH	0.269	0.217	0.379	0.225	0.236	0.529	0.538	0.321	0.368
Av.I	0.429	0.218	0.194	0.571	0.333	0.463	0.250	0.500	0.164
Abs	0.580	0.539	0.260	0.334	0.568	0.290	0.334	0.447	0.334

**Table 4:** An example for calculating the Cochran’s test statistic.

Test A	Test B	D = A–B	D^2^
4.91	4.97	− 0.06	0.0036
4.87	4.95	− 0.08	0.0064
4.87	4.96	− 0.09	0.0081
4.88	4.93	− 0.05	0.0025
4.89	4.94	− 0.05	0.0025
4.88	4.95	− 0.07	0.0049
4.89	4.94	− 0.05	0.0025
4.89	4.94	− 0.05	0.0025
4.91	4.96	− 0.05	0.0025
4.91	4.94	− 0.03	0.0009
Summation of squared difference		*∑D*^*2*^	0.036
Maximum squared difference		*D*^*2*^_*max*_	0.0081
Cochran’s test statistic		C_*Cochran*_	0.225

### Stability of different concentration of PVP-I solution

**Figure 1:**
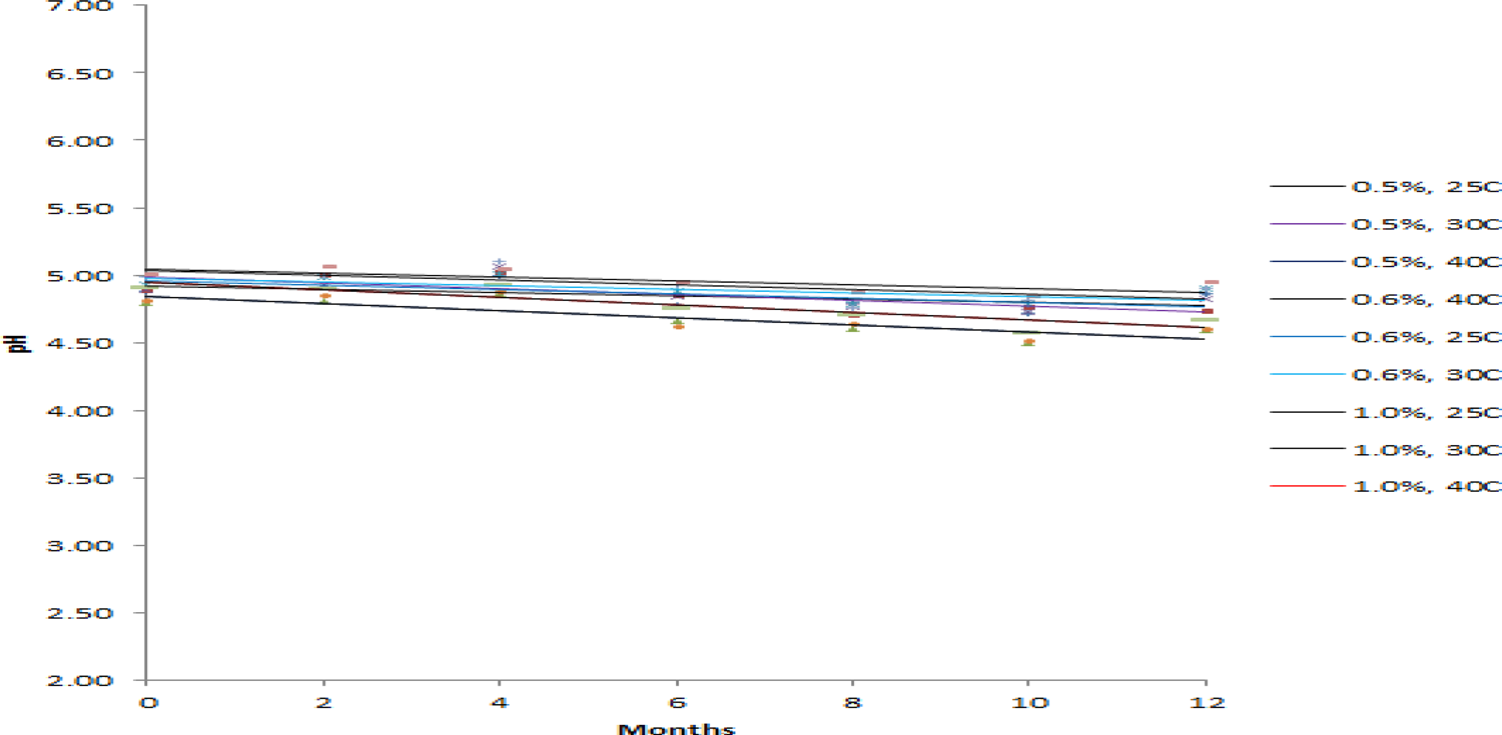
Effect of Storage Condition on pH of PVP-I solutions.

**Figure 2:**
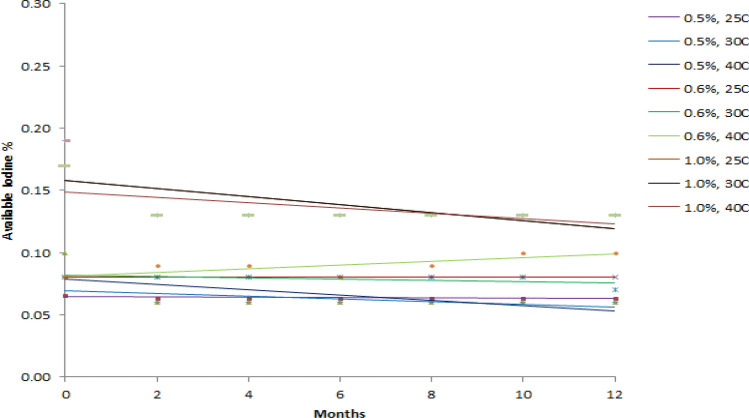
Effect of Storage Condition on Available Iodine of PVP-I solutions.

**Figure 3:**
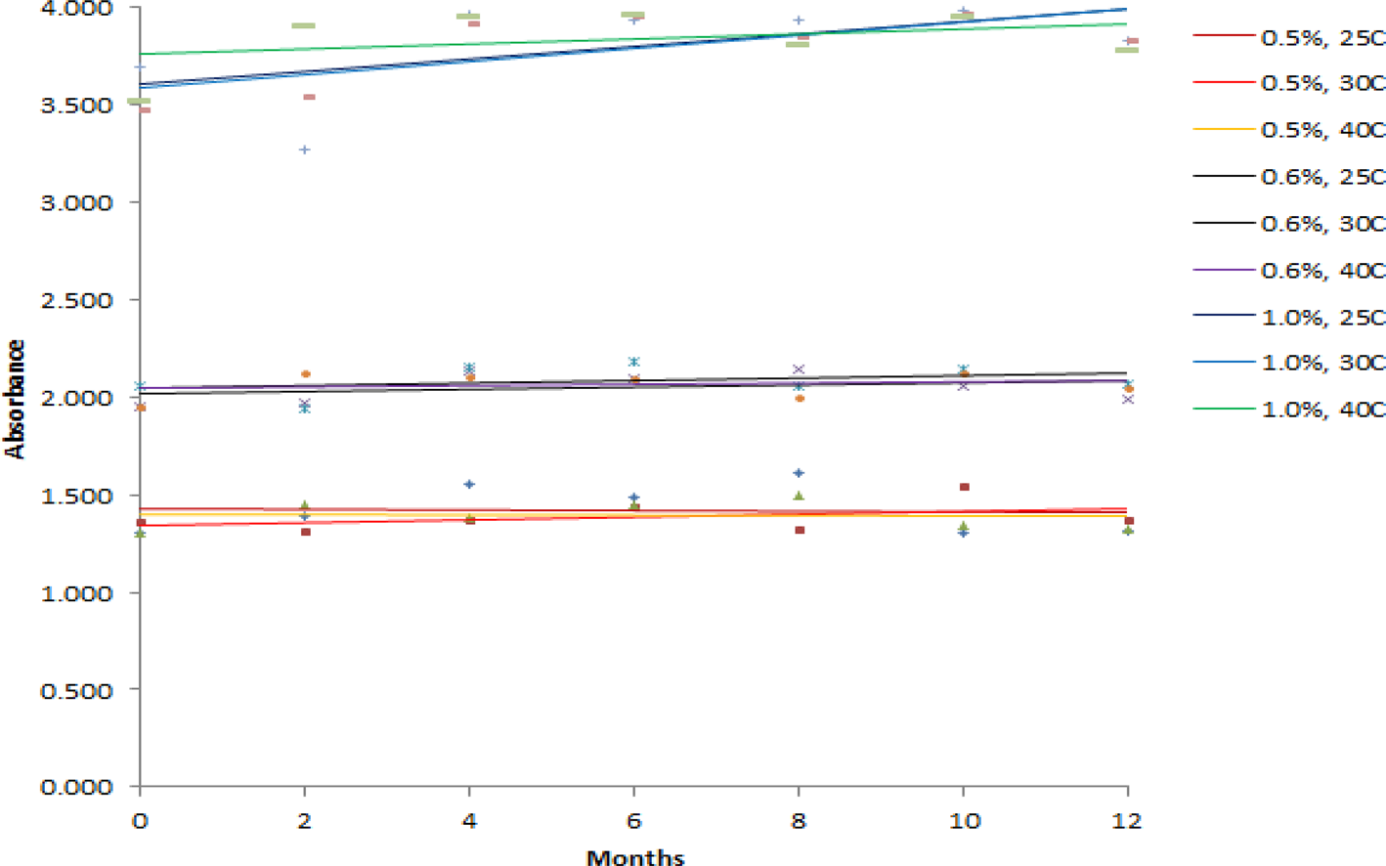
Effect of Storage Condition on Absorbance (at 368 nm) of PVP-I solutions.

Stability of 0.5, 0.6 and 1.0% PVP-I solution were assessed at 25 °C (60%RH), 30 °C (65%RH) and 40 °C (75%RH) based on the pH, Available Iodine % and absorbance at 368 nm of 10 times diluted solution. Regression line for each of the parameter of each prepared solution at above mentioned storage condition was constructed for the period of 12 months with 2 months interval (Figs. 1, 2, and 3). Regression line is generally constructed for graphical observation of the investigated data collected over a period of time which helps to find out any sort of instability quickly at a glance. This approach may often mislead in assessing the stability of a product. Hence, a more reliable approach like one way ANOVA has been introduced where statistical significant difference is observed between initial and final data. For any product to be considered as stable, there should be no significant differences between starting and ending time. Hence, the prepared PVP-I solution that has *p* value > 0.05 for all of the studied parameter (pH, available iodine and absorbance) should be considered stable. The *p* value for each parameter at the studied storage condition is shown in Table 5.

**Table 5:** *p* value for each parameter between initial and final data set.

Sol^n^	0.5% PVP-I			0.6% PVP-I			1.0% PVP-I		
Storage Cond	25 °C (60% RH)	30 °C (65% RH)	40 °C (75% RH)	25 °C (60% RH)	30 °C (65% RH)	40 °C (75% RH)	25 °C (60% RH)	30 °C (65% RH)	40 °C (75% RH)
pH	0.13	0.0002	0.00002	0.06	0.15	0.0002	0.00007	0.0001	0.000001
Av.I	0.33	0.06	0.004	0.55	0.02	0.03	0.00005	0.00002	0.0006
Abs	0.87	0.8	0.36	0.19	0.75	0.05	0.03	0.0005	0.0001

From Fig. 1, it can be seen that the pH of all prepared solution at every storage condition steadily decreases except for 0.5% PVP-I solution at 25 °C and 0.6% PVP-I solution at 25 °C and 30 °C. As for available iodine and absorbance at 368 nm of 10 times dilution, it can be seen from Figs. 2 and 3 that, the available iodine of 0.5% PVP-I solution at 25 °C and 30 °C and 0.6% PVP-I solution at 25 °C remains unchanged; the absorbance of 0.5% PVP-I solution at 25 °C, 30 °C and 40 °C and 0.6% PVP-I solution at 25 °C and 30 °C changes negligibly. These findings from the figures are also supported by the *p* value shown in Table 5. In light of Table 5 and Figs. 1, 2, and 3 it can be said that 0.5% and 0.6% PVP-I solution is stable for 12 months if stored at 25 °C (60%RH). As seen from Table 5 and Figs. 1, 2 and 3, 1.0% PVP-I solution is not stable at any of the storage condition. A strong negative correlation was found (Table 6) between available iodine and absorbance at all the storage condition for 1.0% PVP-I solution which might be the cause of instability of the said solution.

**Table 6:** Correlation between pH, available iodine and absorbance.

1.0% PVP-I, 25C	pH	Av.I	Abs	1.0% PVP-I, 30C	pH	Av.I	Abs	1.0% PVP-I, 40C	pH	Av.I	Abs
pH	1			pH	1			pH	1		
Av.I	0.4	1		Av.I	0.3	1		Av.I	0.4	1	
Abs	− 0.3	− 0.7	1	Abs	− 0.6	− 0.7	1	Abs	− 0.3	− 0.9	1

### Measurement uncertainty estimation of 0.5% and 0.6% PVP-I solution

**Figure 4:**
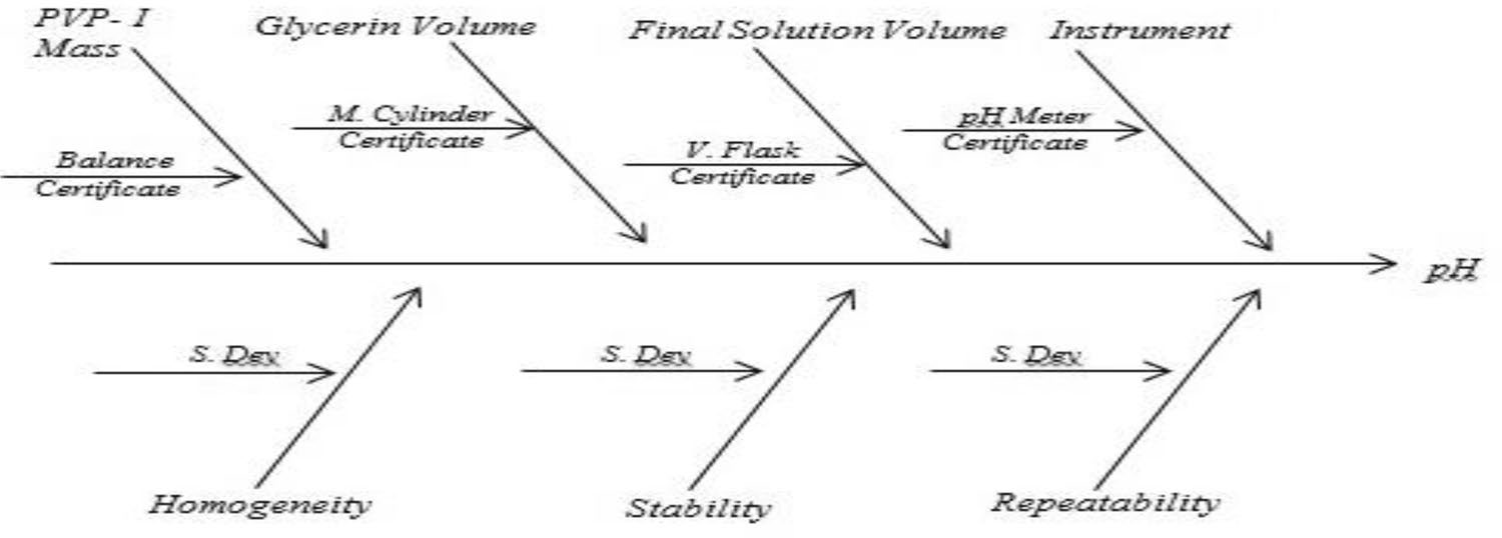
Ishikawa diagram in identifying uncertainty associated in measuring pH of PVP-I solution.

**Figure 5:**
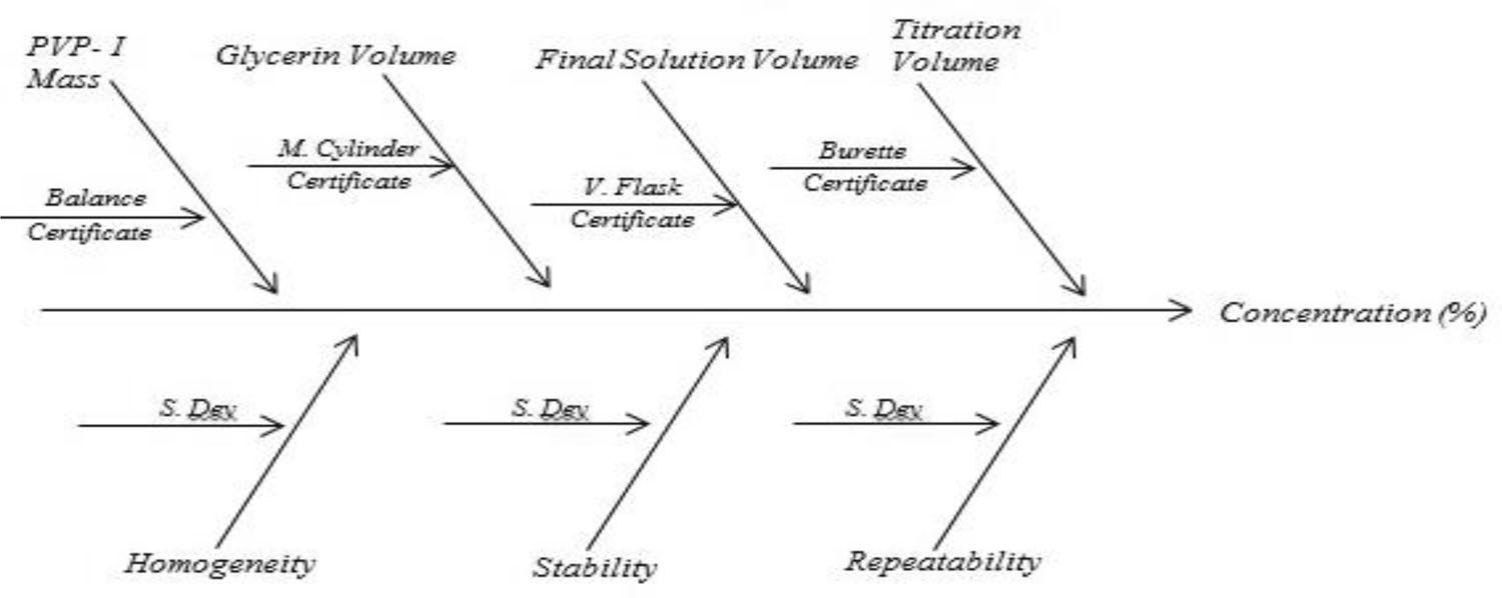
Ishikawa diagram in identifying uncertainty associated in measuring available iodine of PVP-I solution.

**Figure 6:**
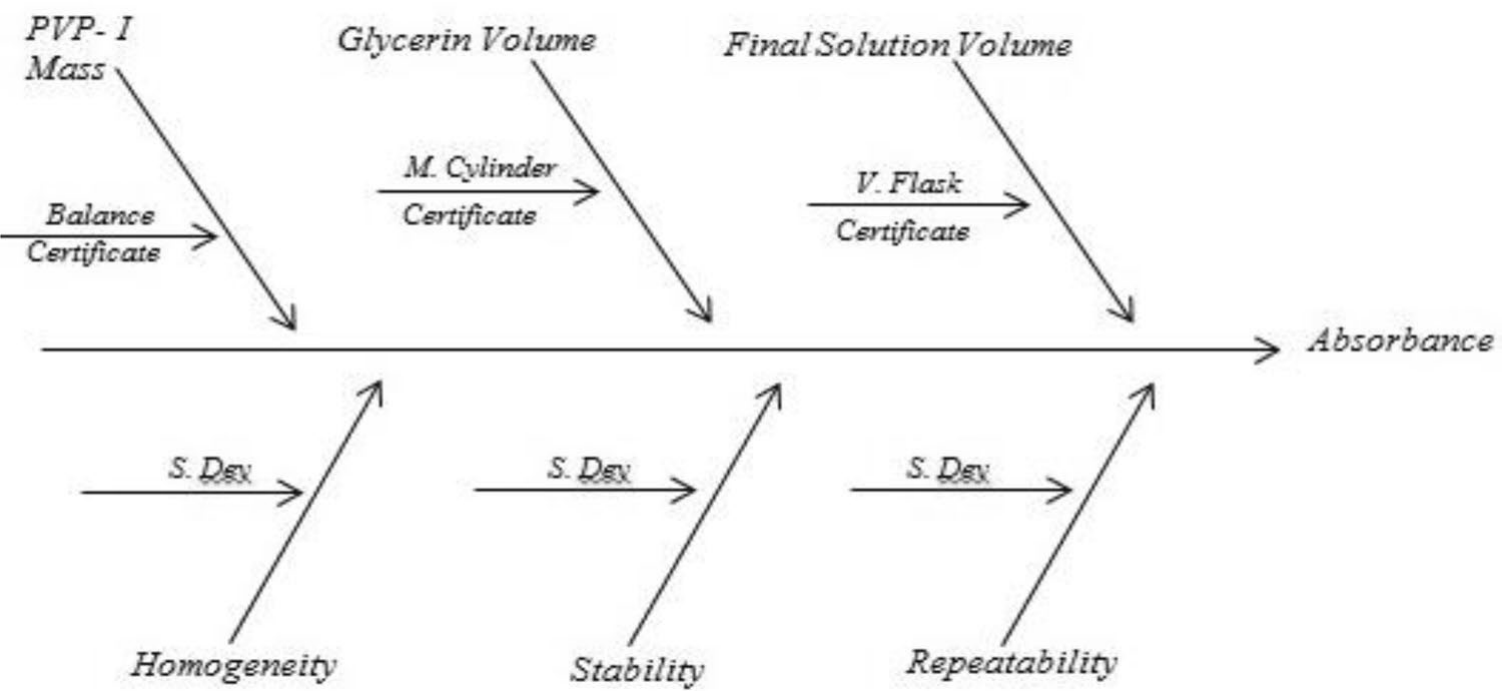
Ishikawa diagram in identifying uncertainty associated in measuring absorbance (at 368 nm) of PVP-I solution.

**Figure 7:**
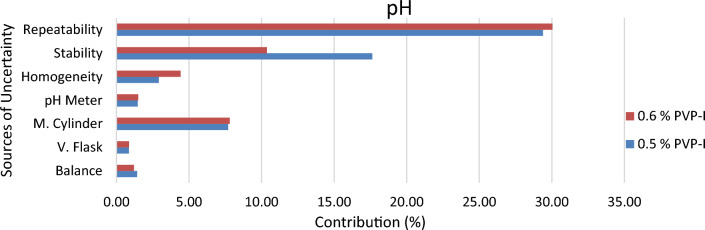
Contribution of Uncertainty Sources in estimating measurement uncertainty for pH of PVP-I solution.

**Figure 8:**
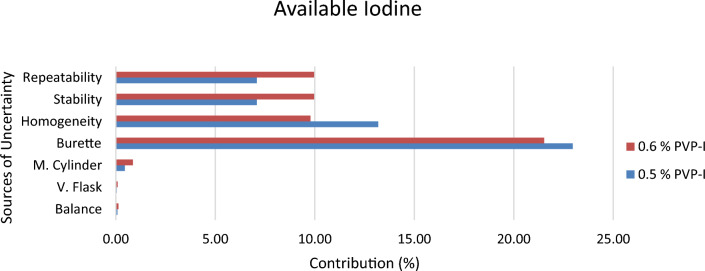
Contribution of Uncertainty Sources in estimating measurement uncertainty for Available iodine of PVP-I solution.

**Figure 9:**
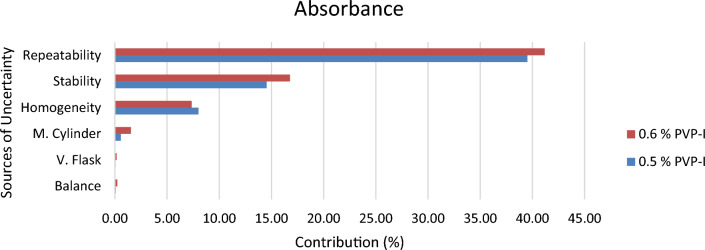
Contribution of Uncertainty Sources in estimating measurement uncertainty for absorbance (at 368 nm) of PVP-I solution.

Since 0.5% and 0.6% PVP-I solution at 25 °C were found to be stable from stability assessment, uncertainty of only these solutions at stable condition were estimated. In order to estimate the uncertainty, an uncertainty budget is needed to be prepared first. Based on source of uncertainty and scope of calibration laboratory the uncertainty budget is prepared. The Ishikawa diagram/fish bone diagram also known as cause and effect diagram is designed in Figs. 4, 5, and 6 to identify the uncertainty associated in measuring pH, available iodine and absorbance of 0.5% and 0.6% PVP-I solution^27^. The uncertainty budget with the expanded uncertainty calculated using Eqs. (1), (2), and (3) for the said parameters of 0.5% and 0.6% PVP-I solution is tabulated in Tables 7, 8, and 9 and contribution of sources of uncertainty in estimating measurement uncertainty is shown in Figs. 7, 8, and 9. It has been seen that repeatability contributed the most in estimating measurement uncertainty for pH and absorbance and stability the second most contributor for both of these cases. This might be because of the standard deviation which was considered as the uncertainty for repeatability and stability. Whereas for the case available iodine, uncertainty from the burette calibration contributed the most.

**Table 7:** The Uncertainty budget for pH of PVP-I solution.

Sources of uncertainty	Unit	Value (x)	Uncertainty (u(x))	Coverage factor (k) (95% CI)	Relative uncertainty (u(x)/x)
0.5% PVP-I solution					
Balance	g	5	0.002	2	0.0002
V. Flask	mL	1000	0.24	2	0.00012
M. Cylinder	mL	167	0.36	2	0.0011
pH Meter	–	4.86	0.002	2	0.00021
Homogeneity	–	4.896	0.002	–	0.00041
Stability	–	4.86	0.012	–	0.0025
Repeatability	–	4.86	0.02	–	0.0041
				Mean value	4.86
			Combined standard uncertainty		0.024
			Coverage factor (95% CI)		2
			Expanded uncertainty		0.05
0.6% PVP-I Solution					
Balance	g	6	0.002	2	0.00017
V. Flask	mL	1000	0.24	2	0.00012
M. Cylinder	mL	167	0.36	2	0.0011
pH Meter	–	4.82	0.002	2	0.00021
Homogeneity	–	4.919	0.003	–	0.00061
Stability	–	4.82	0.0069	–	0.0014
Repeatability	–	4.82	0.02	–	0.0041
				Mean value	4.82
			Combined standard uncertainty		0.02
			Coverage factor (95% CI)		2
			Expanded uncertainty		0.04

**Table 8:** The Uncertainty budget for available iodine of PVP-I solution.

Sources of uncertainty	Unit	Value (x)	Uncertainty (u(x))	Coverage Factor (k) (95% CI)	Relative Uncertainty (u(x)/x)
0.5% PVP-I solution					
Balance	g	5	0.002	2	0.0002
V. Flask	mL	1000	0.24	2	0.00012
M. Cylinder	mL	167	0.36	2	0.0011
Burette	mL	0.05	0.0054	2	0.054
Homogeneity	%	0.0645	0.002	–	0.031
Stability	%	0.06	0.001	–	0.017
Repeatability	%	0.06	0.001	–	0.017
				Mean value	0.06
			Combined standard uncertainty		0.004
			Coverage factor (95% CI)		2
			Expanded uncertainty		0.01
0.6% PVP-I Solution					
Balance	g	6	0.002	2	0.00017
V. Flask	mL	1000	0.24	2	0.00012
M. Cylinder	mL	167	0.36	2	0.0011
Burette	mL	0.1	0.0054	2	0.027
Homogeneity	%	0.0815	0.001	–	0.012
Stability	%	0.08	0.001	–	0.012
Repeatability	%	0.08	0.001	–	0.012
				Mean value	0.08
			Combined standard uncertainty		0.003
			Coverage factor (95% CI)		2
			Expanded uncertainty		0.01

**Table 9:** The Uncertainty budget for absorbance (at 368 nm) of PVP-I solution.

Sources of Uncertainty	Unit	Value (x)	Uncertainty (u(x))	Coverage factor (k) (95% CI)	Relative uncertainty (u(x)/x)
0.5% pvp-i solution					
Balance	g	6	0.002	2	0.00017
V. Flask	mL	1000	0.24	2	0.00012
M. Cylinder	mL	167	0.36	2	0.0011
Homogeneity	–	1.9465	0.01	–	0.0051
Stability	–	2.047	0.024	–	0.012
Repeatability	–	1.946	0.056	–	0.029
				Mean value	1.302
			Combined standard uncertainty		0.06
			Coverage factor (95% CI)		2
			Expanded uncertainty		0.12
0.6% PVP-I solution					
Balance	g	6	0.002	2	0.00017
V. Flask	mL	1000	0.24	2	0.00012
M. Cylinder	mL	167	0.36	2	0.0011
Homogeneity	–	1.3242	0.02	–	0.015
Stability	–	1.423	0.039	–	0.027
Repeatability	–	1.302	0.097	–	0.074
				Mean value	1.946
			Combined standard uncertainty		0.11
			Coverage factor (95% CI)		2
			Expanded uncertainty		0.22

### Application of PVP-I solution on oro-nasopharings against novel SARS-CoV-2 virus

**Figure 10:**
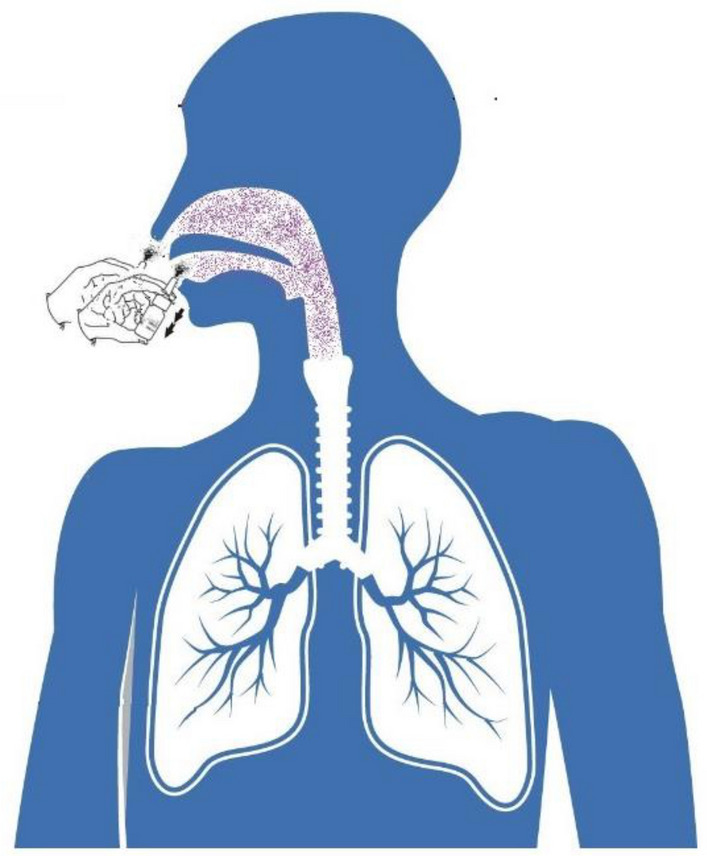
Application of PVP-I solution on oro- nasopharings.

A clinical trial on human has already begun in three hospitals of Bangladesh where these formulated PVP-I solutions (0.5–0.6%) were being applied on oropharings and nasopharings of RT-PCR confirmed COVID-19 patients to assess the viricidal effect of PVP-I against SARS-CoV-2. Two puffs in nose and three puffs in mouth (each puff contains about 80 µL solution) were applied as shown in Fig. 10. Ethical approval was taken from respective authority and a written consent was also taken from the patient who was under the trial. The clearance of SARS-CoV-2 was tested after single time application of formulated PVP-I solution and compared with the corresponding controls. The results were found to be promising so far. More than 80% were found to be negative after single application of formulated PVP-I solution. Detailed results with statistics will be published as soon as the trial is complete.

## Conclusion

This study demonstrated that the prepared 0.5% and 0.6% PVP-I formulation is stable and sufficiently homogeneous at 25 °C (60%RH). Both of the solutions were found to be stable during the studied period of a year. Further stability study will be carried out to investigate whether these solutions will be stable for another year. Measurement uncertainty of these two formulations were estimated and found to be as follows, pH: 4.86 ± 0.05, Available Iodine: 0.06 ± 0.01% & absorbance (at 368 nm): 1.032 ± 0.12 for 0.5% PVP-I solution; and pH: 4.82 ± 0.04, Available Iodine: 0.08 ± 0.01% & absorbance (at 368 nm): 1.946 ± 0.22 for 0.5% PVP-I solution. The clinical trial that is undergoing using these two formulations after single application on oro-nasopharings against novel SARS-CoV-2 Virus showed promising result so far. The formulated solutions may require some modification or development if significant number of discomfort or irritation is reported by the patients during the clinical trial. In that case another study will be proposed to investigate the effect of modification or development on the original formulation.
